# Facile-Solution-Processed Silicon Nanofibers Formed on Recycled Cotton Nonwovens as Multifunctional Porous Sustainable Materials

**DOI:** 10.3390/ma17020412

**Published:** 2024-01-14

**Authors:** Muhammad Shoaib, Hafsa Jamshaid, Rajesh Kumar Mishra, Mumtaz Ali, Vijay Chandan, Viktor Kolar, Shabnam Nazari, Akshat TM, Petr Jirku, Miroslav Muller, Tatiana Alexiou Ivanova

**Affiliations:** 1 School of Engineering and Technology, National Textile University, Faislabad 37610, Pakistan; ranashoaib.shoaib@gmail.com (M.S.); hafsa@ntu.edu.pk (H.J.); 2 Department of Material Science and Manufacturing Technology, Faculty of Engineering, Czech University of Life Sciences Prague, Kamycka 129, Suchdol, 165 00 Prague, Czech Republic; vijay@tf.czu.cz (V.C.); vkolar@tf.czu.cz (V.K.); jirkup@tf.czu.cz (P.J.); muller@tf.czu.cz (M.M.); 3 Department of Sustainable Technologies, Faculty of Tropical AgriSciences, Czech University of Life Sciences Prague, Kamycka 129, Suchdol, 165 00 Prague, Czech Republic; nazari@ftz.czu.cz (S.N.); ivanova@ftz.czu.cz (T.A.I.); 4 Department of Machine Design and Mechanism, Faculty of Mechanical Engineering, Technical University of Liberec, 46 117 Liberec, Czech Republic; akshattm93@gmail.com

**Keywords:** recycling, cotton waste (CW), sustainability, porous nonwovens, self-cleaning, antibacterial, surface modification

## Abstract

Limited efficiency, lower durability, moisture absorbance, and pest/fungal/bacterial interaction/growth are the major issues relating to porous nonwovens used for acoustic and thermal insulation in buildings. This research investigated porous nonwoven textiles composed of recycled cotton waste (CW) fibers, with a specific emphasis on the above-mentioned problems using the treatment of silicon coating and formation of nanofibers via facile-solution processing. The findings revealed that the use of an economic and eco-friendly superhydrophobic (contact angle higher than 150°) modification of porous nonwovens with silicon nanofibers significantly enhanced their intrinsic characteristics. Notable improvements in their compactness/density and a substantial change in micro porosity were observed after a nanofiber network was formed on the nonwoven material. This optimized sample exhibited a superior performance in terms of stiffness, surpassing the untreated samples by 25–60%. Additionally, an significant enhancement in tear strength was observed, surpassing the untreated samples with an impressive margin of 70–90%. Moreover, the nanofibrous network of silicon fibers on cotton waste (CW) showed significant augmentation in heat resistance ranging from 7% to 24% and remarkable sound absorption capabilities. In terms of sound absorption, the samples exhibited a performance comparable to the commercial standard material and outperformed the untreated samples by 20% to 35%. Enhancing the micro-roughness of fabric via silicon nanofibers induced an efficient resistance to water absorption and led to the development of inherent self-cleaning characteristics. The antibacterial capabilities observed in the optimized sample were due to its superhydrophobic nature. These characteristics suggest that the proposed nano fiber-treated nonwoven fabric is ideal for multifunctional applications, having features like enhanced moisture resistance, pest resistance, thermal insulation, and sound absorption which are essential for wall covers in housing.

## 1. Introduction

According to the US Environmental Protection Agency (US EPA), every year, 9.5 million metric tons of apparel and textiles end up in landfills in the USA alone, and less than 1% is recycled back into new materials [1]. However, it was estimated that more than 80% of this cotton waste (CW) is suitable for mechanical recycling into new textile materials [2]. Using recycled cotton has the potential to reduce wastage of water, energy, pesticides, and dyeing chemicals, and reduce landfill. Global interest in CW as an underutilized resource has been steadily growing, driven by its status as the second most produced fiber after polyester. Therefore, exploring and improving techniques for transforming CW into valuable commodities and effectively incorporating them into waste management practices is imperative. This approach not only contributes to the conservation of resources but also strengthens the concept of a circular economy and facilitates sustainable development [3,4,5,6,7]. Recycling CW throughout all stages of its production and usage can significantly minimize its environmental impact, reducing the agricultural carbon footprint associated with its cultivation [8].

Using nonwovens from CW in building insulation can potentially offset the carbon footprint from material’s usage and disposal, contributing to energy-efficient and acoustically insulated buildings [9,10]. Furthermore, the versatility of these recycled materials is evident in their growing presence in the nonwoven fabric sector, which is increasingly recognized for its pivotal role in sound absorption applications, offering additional attributes such as flexibility, durability, and softness [11,12]. Several studies have been conducted to explore the potential of using several other natural fibers, such as palm, kenaf, coconut, coir, and others, in the development of nonwoven materials specifically designed for acoustic applications [13]. Researchers highlighted the use of recycled fibers in the production of nonwovens as a viable and economically advantageous option for manufacturing acoustic materials [14]. Moreover, recycled polypropylene and polyester absorb sound at mid-range frequencies more efficiently owing to their inhomogeneity and micro voids [15]. However, it was found that acoustic performance is affected by the fiber type, fineness, density, bonding methods, airflow resistivity, and porosity of nonwovens [16,17]. Increasing the proportion of fibers with smaller diameters in a given layer (which decreases the air permeability) or increasing the thickness of a layer containing a given distribution of fiber diameters improves the sound absorption. An average sound adsorption coefficient (SAC) of 0.95 was found in nonwoven Kenaf-fiber-based materials for frequencies of 1250 Hz. Including an air gap between a sound absorbing layer and its rigid backing increases the SAC even at lower frequencies [18]. On the other hand, sound absorption ability also depends on the interior pore architecture, with a greater pore count indicating better absorption [19]. This refers to smaller pores and the bulk porosity of the material. With an increase in the number of micropores which form a network of interconnected channels, the air flow resistivity increases, ultimately increasing the sound absorption. It was reported that recycled polyester nonwovens with lower denier polyester fibers orientated at 90° had better sound insulation as the surface roughness of polyester fibers improved the acoustic performance of composite materials [20]. In the context of thermal insulation materials, the incorporation of recycled cotton and polyester fibers into nonwoven materials proved to be a significant breakthrough. This development is marked by several advantageous features, including its cost-effectiveness, ease of manipulation, and environmental sustainability [21]. The advancements highlight the inherent potential of incorporating recycled wastes into the fabrication of materials that exhibit commendable acoustic and thermal insulation properties. Simultaneously, there has been an increased focus on the development of sound insulators using a variety of recycled fabrics, and investigating their potential in terms of thermal resistance. A thermal conductivity value of (k = 0.033 W/m.K) suggests that such nonwovens possess favorable characteristics that make them suitable for use as insulation materials [22]. Thermal and sound insulation samples manufactured from recycled wool and polyester textile waste were proven to be effective in construction applications [23]. These types of samples present promise as acoustic absorbers, characterized by their absorption coefficients, but not as acoustic insulators. Among technical textile applications, thermal and acoustic insulation is crucial for wall lining and interior design [24]. Chemically bonded nonwovens were produced from recycled cotton/PET for use as thermal insulation in building construction [25]. Recycling natural fibers is the ultimate solution to suppress the accumulation of solid waste, along with reusing the waste for useful purposes. As mechanical recycling is carried out for natural fibers, there is a chance of fiber breakage and shortening of fiber length. The inclusion of short fibers in fine yarn may lead to the material having a lower strength, and so it cannot be utilized for continuous weaving or knitting processes. As an alternative, the simple approach of creating nonwoven fabrics from recycled cotton was adopted. The thickness of the nonwoven samples was changed to achieve a suitable performance, i.e., one comparable to that of commercially available sound-absorbing materials. Furthermore, to enhance the functional aspects, a superhydrophobic finish was applied via a facile process, using eco-friendly and organic solvent-free materials. Conventionally, chlorofluorocarbon-based materials are used for hydrophobic modification (with a contact angle higher than 90°); however, their toxicity has caused a shift toward alternative eco-friendly materials.

However, limited efficiency, durability concerns, moisture absorbance, and bacterial growth are major issues regarding the use of CW nonwovens as building insulators [26]. Therefore, there is a dire need for facile and scalable techniques of producing hydrophobic nonwoven fabrics that are also mechanochemically durable. Their hydrophobic properties might enable it to be a highly versatile material in various domains. Notably, they can find utility in the development of self-cleaning surfaces [27,28,29], water-resistant clothing [30], and anti-corrosion coatings [31].

Previous studies highlight the importance of micro and nano air pores for improving sound absorption and thermal insulation. These are the micropores which form a network of interconnected channels. Closed micropores are important for thermal insulation, whereas a network of interconnected pores which are open at the sound-exposed surface is important for sound absorption. Similarly, a high surface area also assists with the fabrics’ superhydrophobic characteristics, as surface roughness is higher in finer nano-fibrous structures. Despite the high performance of such nanofibers, their scalability limits their practical applications. To address these issues, a facile-solution processing approach has been developed in this work, for the one-step synthesis and coating of silicon polymer nanofibers onto nonwoven fabrics from CW. As a result, a unique adaptable strategy for the creation of robust superhydrophobic surfaces, without using organic solvents and toxic compounds, has been achieved. The prepared nonwovens were compared with a commercial sample. The surface-modified sample exhibited significant advantages and potential in self-cleaning and antibacterial properties, sound absorption, and thermal resistance. This strategy offers a cost-efficient, simple, and effective way to create superhydrophobic surfaces, which are useful in multifunctional applications, such as wall linings, curtains, and protective gear.

Silicon treatment effectively imparts superhydrophobic properties to nonwoven fabrics, increasing their versatility across a broad range of applications [32]. By applying silicon nanofibers, the wettability of the fabric decreases, creating a water-repellent barrier while maintaining breathability and flexibility. These nanofiber-modified fabrics are valuable in the medical, hygiene, filtration, and protective clothing industries due to their enhanced liquid barrier performance, durability, microbial growth resistance, and self-cleaning performance [33]. For this purpose, silicon-based nanofibers were developed via facile chemistry, serving both as a binder and surface modifying agent. The acoustic and thermal insulation performances of the fibers were studied in detail.

## 2. Materials and Methods

### 2.1. Materials

Cotton fiber waste was procured from local textile factories in Pakistan to develop nonwoven fabrics. Mechanical shredding and opening of CW were carried out using commercial mechanical opening systems. The average 50% and 2.5% fiber span length measured after mechanical recycling is given in Table 1, which is suitable for the nonwoven manufacturing process. The values depict (a) 50% span length (50% of all the fibers have this length or higher) and (b) 2.5% span length (2.5% of all the fibers have this length or higher). These definitions of span length are as per the norms in fiber science and textile engineering terminologies. These are standard parameters explaining the length of fibers to be processed through textile processing machinery, e.g., carding and nonwovens needle punching.

Room temperature vulcanizing (RTV) silicon^®^ from GMSA, Faisalabad, Pakistan, was used for the formation of nanofibers, and deionized water was used as the carrier. RTV silicon is a colorless transparent liquid, with a crosslinking agent, which acts as a catalyst for room temperature curing, maintains its long-term flexibility in the temperature range of 60–200 °C, has excellent electrical properties and chemical stability, water resistance, and resistance to weathering. It is widely used in electronics, semiconductor, automotive, machinery, textiles, plastics, printing, and construction applications. The properties of RTV silicon are provided in Table 2.

### 2.2. Methods

#### 2.2.1. Fabrication of CW Nonwoven Fabrics

**Figure 1:**
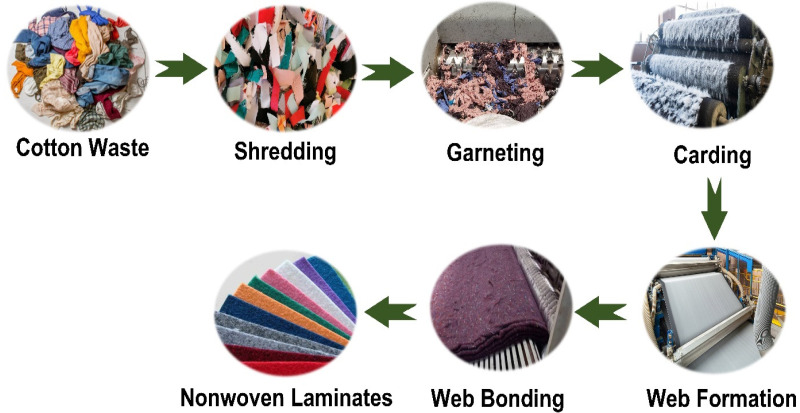
Illustrative process sequence to fabricate nonwoven laminates utilizing CW.

CW, including scraps and fabrics, was first sorted and cleaned to remove contaminants like dirt, dust, and debris. Further, it was subjected to a shredding process which converted it into a homogenous fibrous mass. A fiber-opening process was carried out, which involved mechanical devices like tearing and garneting machines. These operations separated and opened the fibers, preparing them for further processing. The opened fibers were then carded to disentangle and align them into a uniform, loose, fibrous web. The carded fibers were layered to form a web with a desired thickness and weight. The layers of the web were then bonded to create a nonwoven laminate by using the needle punching technique, in which barbed needles pushed the fibers through the thickness to entangle them. A finishing process involving calendaring was carried out to enhance compactness/density and uniform appearance. An illustrative process sequence is shown in Figure 1.

#### 2.2.2. Formation of Silicon Nanofibers on the Nonwoven Surface

**Figure 2:**
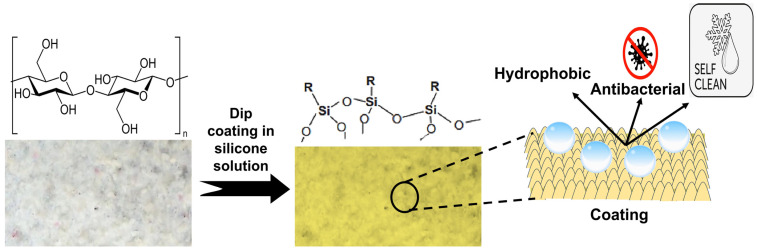
Overview of silicon treatment and its corresponding effects on the properties of nonwoven laminates.

In the present research, room temperature vulcanizing (RTV) silicon^®^ from GMSA, Faisalabad, Pakistan, was used for the formation of nanofibers. The silicon was plasma-treated under oxygen gas for five minutes to allow easy dispersion in water via vigorous stirring. A silicon-in-water emulsion with a concentration of 5 wt% was formed, which was applied to the nonwoven fabrics by using a pad–dry–cure process (as shown in Figure 2) to effectively modify their surface. Due to plasma treatment, silicon was dispersed in water; however, heat treatment carried out during the curing process makes it hydrophobic again. Microscopic analysis confirmed the presence of nanosized silicon fibers on the surface of CW nonwovens. The add-on was found to be about 1.5 wt%, which also corresponds to previous literature [31,32,33]. Figure 2 illustrates the difference in surface chemistry after silicon treatment and the functionalities induced after the treatment of nonwoven fabrics.

This study examined five different types of nonwoven laminates (designated as types A, B, C, D, and M). Sample M is the commercial nonwoven material available in the market for sound insulation purposes. However, it was available only in the given thickness. Thickness is considered to be a major parameter which governs overall porosity and thus determines the thermal and acoustic performance. Therefore, experimental samples A, B, and C were created with the same type of fiber content but with different thicknesses. These samples were mechanically finished by heat treatment and calendaring. All of them were treated with silicon via the pad–dry–cure process to effectively modify their surface. Owing to their similar construction, the parameters of the treated samples were very close to each other. Therefore, only one type of treated sample, D, is considered in this investigation. Table 3 shows the nonwoven samples investigated in the study.

### 2.3. Characterizations

#### 2.3.1. Surface Morphology and Structural Characteristics

Scanning electron microscopy (SEM, JEOL JSM 6700F, EVISA, Helioparc, 64000 Pau, France) was used for high resolution topography of cotton fibers and silicon nanofibers formed. Fabric samples were sputter coated with gold for 1 min under vacuum. For the elemental composition of treated samples, X-ray photoelectron spectroscopy (XPS) was performed using a multilab ESCA2000 system-version 2023 (Thermo Fisher Scientific, Waltham, MA, USA). For the photoexcitation, a mononchromatic Al Kα source was utilized with an energy step size of 0.05 eV. Fourier transform infrared (FTIR) spectrometry was performed in the attenuated total reflectance (ATR) mode, using Nicolet TM iSTM, for the analysis of functional groups.

#### 2.3.2. Physical Characterization

Physical characteristics can impact the performance of a nonwoven fabric depending on its composition, the manufacturing process, and any post-processing or finishing treatments applied. The physical characteristics, such as thickness, porosity and GSM (grams per square meter), of the nonwoven fabrics were measured using standard procedures.

GSM is a critical parameter in assessing the areal density of fabrics, indicating the fabric’s weight per unit of area. It is routinely measured using a GSM cutter, a straightforward and reliable tool common in fabric manufacturing industry by using ASTM-D3776 standard [34]. The cutter cuts the fabric into a standard circular shape which is weighed to obtain the value of GSM. This instrument cuts a precise area of fabric to be weighed, yielding the GSM value, thus providing insight into the fabric’s areal density and various performance aspects. Fabric thickness, gauged by the separation between the upper and lower fabric layers, is quantitatively determined using a thickness meter applying a pressure of 8.6 ounces (OZ). Measurements, complying with the ASTM D1777 [35] standard, were conducted within a range of 0.1–25.4 mm by using a digital fabric thickness tester, from (SDL Atlas, South Carolina, USA). The pore size in nonwoven fabrics was quantified using digital microscopy (OPTIKA, Ponteranica, Bergamo, Italy). Air permeability, a vital characteristic of non-woven materials, is influenced by the fabric’s thickness, porosity, and pressure gradient. Air permeability of the nonwoven samples was measured by using air permeability tester by (SDL Atlas, Rock Hill, SC, USA) and standard ASTM D737-18 [36].

#### 2.3.3. Mechanical Characteristics

Mechanical properties are very important features that can influence the material’s durability and functionality. Tear strength is a measure of the ability of a material to resist the propagation of cut marks. Tear strength was measured according to the ASTM D2261-13 [37] using a tear strength tester from (SDL Atlas, SC, USA). Bending resistance in nonwoven fabrics refers to their resistance to deformation under flexural load, which relates to fabric drape and flexibility. It is governed by fiber type, the bonding method, and fabric thickness. A higher bending strength usually implies a stiffer fabric with lower drape. Bending length was measured based on ASTM-D1388 [38] and a bending tester from (SDL Atlas, SC, USA). This involves bending a fabric sample and assessing the force needed for a specific angle of bending. The desired bending strength varies depending on the application, with higher strength favored for dimensional stability, while lower strength is preferred for improved drapes especially in apparel or furnishings.

#### 2.3.4. Acoustic Characteristics of Fabrics

The acoustic performance of nonwoven fabric samples was measured according to ASTM E-1050 [39] using the impedance tube method. The acoustic qualities of textiles, particularly their impedance matching and transmittance traits, are evaluated using the Brüel and Kjær impedance tube technique. The device consists of two impedance tubes for sound absorption testing within the frequency range 50 Hz–6.4 kHz. A larger tube (100 mm in diameter) and a smaller tube (29 mm in diameter) can be set up for measuring the sound absorption in a low-frequency range from 50–1600 Hz and a high-frequency range 500–6400 Hz, respectively. The sound absorption values of lower common frequencies (from 50 to 1600 Hz) mainly come from the larger tube and the higher common frequencies (up to 6400 Hz) mainly come from the smaller tube. The low-frequency range was chosen to avoid inaccuracies caused by structural vibrations or phase mismatch.

This method measures the material’s reflection coefficient, enabling the absorption coefficient to be computed, by using a 100 mm diameter tube, two transducers, and digital spectrum statistical software. The fabric samples were put inside the test tube adjacent to the sound source. The sound intensity and frequency were measured using a two-channel fast Fourier transform (FFT) analyzer, and the normal incidence absorption coefficients were averaged over frequency. Ten samples from each category were tested.

The sound absorption coefficient SAC (α) indicates how much of the sound is absorbed by the material. When sound waves propagate into a media one part of the input sound energy *W_i_* [J] is transformed into heat (*W_q_* [J]), a second part is reflected back (*W_r_* [J]), and a third part is transmitted through the insulation layer (*W_t_* [J]). Thus, the methodology involves the measurement of input and output sound energy which already includes the influence of thickness. The sound absorption coefficient SAC (α) can be defined as:

(1) $$\alpha =1-\frac{W_{r}}{W_{i}}=\frac{W_{q}+W_{t}}{W_{i}}$$

The normal incidence sound absorption coefficient of nonwovens was determined to be a function of the sound frequency. The average *SAC* of nonwoven samples were calculated using the following equation:

(2) $$SAC=\frac{\alpha_{250Hz\;}+\alpha_{500Hz\;}+\alpha_{1000Hz\;}+\alpha_{2000Hz\;}}{4}$$

#### 2.3.5. Thermal Resistance

Nonwoven sheets can prevent heat loss due to their relatively high thermal resistance. The thickness, density, composition, and structure of the fibers control the amount of air entrapped, which influences their thermal resistance. Nonwoven materials may be used for insulation in a variety of sectors, including apparel and buildings, because of their insulation related qualities. By evaluating the thermal conductivity, which often involves a test using hot and cold plates, thermal resistance is commonly determined. To choose and optimize nonwoven sheets for thermal insulation requirements, it is essential to evaluate this property. The thermal conductivity and resistance of the nonwoven sheets were measured according to ISO 11092:2014 [40] using an Alambeta thermal insulation tester from (Sensora, Liberec, Czech Republic).

#### 2.3.6. Anti-Bacterial Characteristics

Qualitative analysis of antibacterial performance was performed using Escherichia coli (*E. coli*) and Staphylococcus aureus (*S. aureus*) strains using test standard AATCC TM147 [41]. A single colony of *S. aureus* bacteria and the nonwoven samples were added to the broth, and it was incubated at 37 °C for 24 h. The growth behavior of bacteria on silicon-nanofibers-modified nonwoven and pristine nonwoven materials was compared.

## 3. Results and Discussion

### 3.1. Morphological and Compositional Characterization

Scanning electron microscopy (SEM) was used to analyze the surface morphology of nonwoven fabrics before and after surface modification.

**Figure 3:**
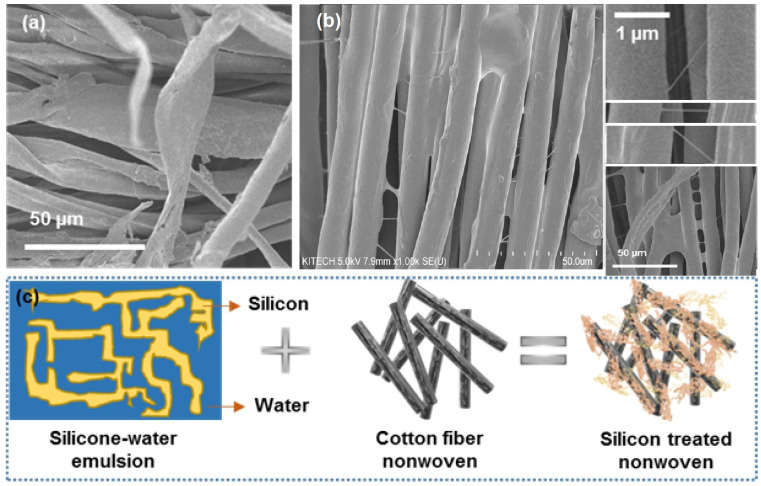
SEM of (**a**) untreated waste cotton nonwoven and (**b**) silicon-modified cotton nonwoven. (**c**) Mechanism of silicon nanofibers formation on the surface of cotton waste nonwovens.

Figure 3a shows the nonwoven fabric’s structure without any coating, where the twisted ribbon-like structure of bare cotton fibers can be observed clearly. On the other hand, after being modified with silicon (Figure 3b), additional interconnected networks of nanofibers can be observed between the cotton fibers. In addition, there exists a coating of silicon on the individual cotton fibers; therefore, after coating, the fibers are observed to have a rounded morphology. Comparing the diameter of the cotton fibers before and after coating, a significant difference was not observed, showing that a very fine and thin film was formed after coating. The high-resolution insert in Figure 3b shows a fine, interconnected, nano-fibrous network, in which the individual nanofibers have a diameter of less than 50 nm. The formation of an interconnecting, nano-fibrous network between the cotton fibers helps in increasing the roughness of microfibers at the nano-level. This results in a hierarchical rough structure at the micro scale. Such micro-nano roughness offers the entrapment of more micro air pockets and a high contact angle for water droplets. Ultimately, this provides the desired level of hydrophobicity [42,43,44,45]. Additionally, these flexible connections between nanofibers are suitable for enhancing the mechanical stability of the nonwoven materials [46].

The mechanism of silicon nanofiber formation is illustrated in Figure 3c. The silicon forms fibrillated nanostructures in water. This emulsion is stabilized in water due to the plasma treatment of silicon, which makes it slightly hydrophilic in nature [47,48,49]. Plasma treatment generates oxygenated functional groups, responsible for the dispersion of silicon under stirring. However, upon heat treatment, these newly formed oxygenated bonds are removed, and the silicon retains its hydrophobic nature [50]. It is important to note that a vigorous stirring with a mechanical shear mixer is essential to achieve a fine dispersion, which is responsible for the formation of the nano-fibrous network. Otherwise, coarser structures are formed, which reduces the micro porosity and increases air permeability of the nonwoven fabric structure. Combining the fine emulsion of silicon in water and the nonwoven fabric resulted in a modified nonwoven fabric structure, schematically shown in Figure 3c. Compared to the electrospun nanofibrous structure, the proposed nanofibrous structure is embedded inside the fabric’s structure, having a stronger interface and adhesion with cotton fibers. Therefore, higher mechanical stability is expected for the proposed system, as required for practical applications. Robustness aaside, the proposed method is faster, continuous, and scalable in continuous industrial-scale processes, unlike electrospinning of nanofibers which have limited production yields.

**Figure 4:**
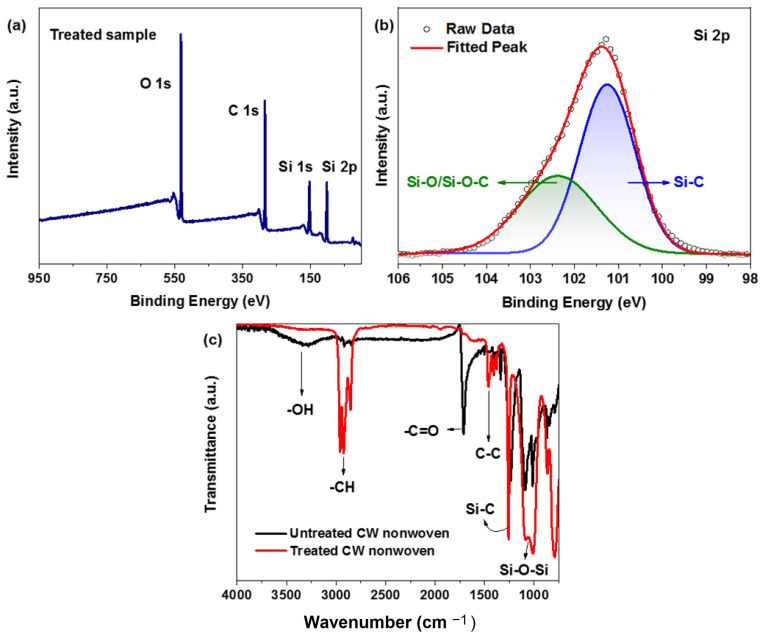
Analysis of the chemical composition of the treated sample using (**a**,**b**) XPS and (**c**) FTIR.

The chemical composition and structure of the final product were analyzed using XPS, as shown in Figure 4a,b. Elemental analysis of XPS confirmed the presence of three constituents: silicon, carbon, and oxygen, centered at a binding energy of 103, 285, and 530 eV, respectively. Silicon showed two peaks related to the 1s and 2p energy levels, as labeled in Figure 4a. These elements are from the co-existence of the cotton fibers and silicon nanofibers, as well as the coating. Specifically, the atomic percentage of Si 2p, C 1s, and O 1s was around 25%, 45%, and 28%, respectively [51]. The major carbon content was due to the nearly uniform surface coating of the nonwoven fabric structure. A high-resolution analysis of silicon is given in Figure 4b, which shows the core skeleton of silicon polymer coating deconvoluted in two peaks: Si-O bonds at 102.5 eV [47] and Si-C bonds at 101.2 eV [52,53]. The integrated area under the curve for both peaks was 60% and 40% for Si-C and Si-O bonds, respectively, showing the characteristic structure of a silicon polymer.

FTIR analysis was performed in ATR mode in order to analyze the functional groups of the treated and untreated samples. The functional groups of both samples are highlighted in Figure 4c, revealing a significant difference in functional groups before and after the formation of silicon nanofibers. In particular, untreated nonwoven CW showed –OH, C=O, and COOH bending peaks at 3330, 1700, and 1230 cm^−1^, respectively [54,55]. These oxygenated functional groups impart a hygroscopic and hydrophilic nature to the cotton fiber, as these polar functional groups can form hydrogen bonds with the water molecules [51]. On the other hand, C-H, C-C, Si-C, and Si-O-Si peaks were dominant after treatment with silicon nanofibers, which were centered at 2930, 1450, 1250, and 990 cm^−1^, respectively [52,53,54]. Compared to the peaks for cotton fiber, the additional peaks formed by silicon nanofibers are nonpolar, thus imparting a superhydrophobic nature to the silicon treated CW nonwovens. The combined effect of nonpolar surface chemistry and additional micro roughness imparted by nanofibrous architecture is responsible for the super hydrophobicity of the treated samples.

### 3.2. Physical Characteristics

The physical characteristics of nonwoven fabrics composed of recycled 100% cotton and their average values are presented in Table 4. The samples include a commercial market sample (M), nonwoven samples with different thicknesses (Sample A, B, C), and a surface-modified sample (D). These samples were subjected to physical measurements according to ASTM standards.

#### 3.2.1. Areal Density (GSM)

**Figure 5:**
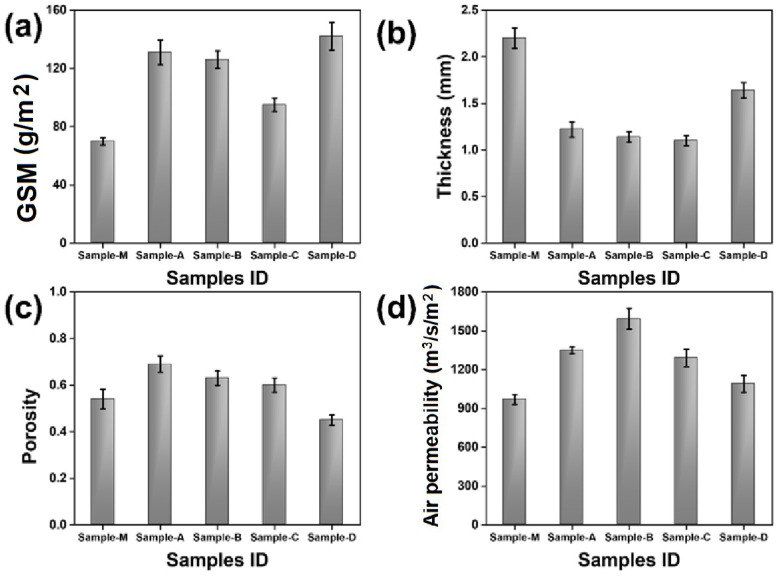
Physical characteristics of nonwovens compared with market sample. (**a**) GSM, (**b**) thickness, (**c**) porosity, and (**d**) air permeability.

To evaluate the quality of a fabric, the ratio of its mass to its area, or GSM, must be determined. Fabrics with a higher GSM indicate that the fibers are packed closer together, making the material thicker. Therefore, nonwoven materials with a higher GSM value tend to be denser. Figure 5a indicates that sample D had slightly higher GSM (by 7.74%) because of the increase in the weight of nonwoven fabric after treatment with silicon. Compared to this, the GSM values of other nonwoven textiles produced were relatively lower due to the larger air gaps between the fibers that result from a less compact arrangement. Sample M has the lowest GSM due to its minimum areal density as shown in Table 4.

#### 3.2.2. Thickness

Figure 5b shows that, due to the integration of numerous layers entangled with each other, the market sample was thicker than the nonwovens developed in the laboratory. However, the prepared nonwoven textiles were thinner because they had relatively fewer layers and the fibers were more compact. The thickness of sample D was higher (by 25.6–32.92%) than the other prepared nonwoven samples due to its surface treatment. When compared to the thickness of other samples, sample M was 40–50% thicker. The higher thickness of sample M is due to minimum entanglements among fibers and a relatively lower density.

#### 3.2.3. Porosity

The characterization of the porosity of a material involves quantifying the ratio of its vacant space, also known as void volume, to its overall total volume. Sample D demonstrates a decrease in porosity because of the surface treatment that effectively sealed its pores as shown in Figure 5c. The lower porosity of sample D is due to empty spaces (pores) being blocked by the silicon solution and by nanoscale fibrils created in the process. The porosity of sample D was lower, by 16.67%, than the other samples developed and 25–35% lower than the market sample M, respectively. The sample M was created by the manufacturer as multilayered nonwoven webs bonded together by needle punching in the thickness direction. Therefore, the orientation of the selected fibers in the thickness direction help in bonding the layers together and result in a relatively denser structure with a lower porosity. These disoriented fibers further hamper the continuity of the porous channels. Therefore, the porosity of sample M was relatively lower due to the presence of many interconnected layers that impede the continuity of pores. The samples A, B, and C were developed with a relatively lose construction. The loose fibers result in a higher porosity as well as a continuity of porous channels. These nonwoven samples were not subjected to any treatment and thus exhibited higher level of porosity due to the presence of loosely arranged fibers inside the fabric structure.

#### 3.2.4. Air Permeability

The air permeability of a fibrous material pertains to its capacity to allow the passage of air. The results shown in Figure 5d indicate that sample M had a lower air permeability (higher air flow resistivity) compared to other nonwoven samples due to entanglements of the multiple layers that block the passage of air while the other samples had decreased fiber entanglement and gaps that permitted air to pass through. However, sample D showed 15.55–19.19% lower air permeability (higher air flow resistivity) than the other nonwoven samples we prepared due to its surface treatment which formed nanofibrous structures and hindered the passage of air. A higher airflow resistivity is necessary for better acoustic insulation.

### 3.3. Mechanical Characteristics

**Figure 6:**
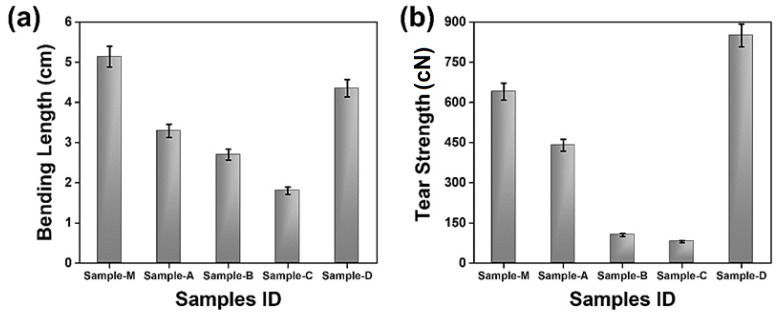
Mechanical performance of nonwovens. (**a**) Bending length; (**b**) tear strength.

The bending lengths and tear strengths of the samples were measured and the results of the developed nonwoven samples were compared with the commercial sample obtained from the market. The results are shown in Figure 6a,b.

#### 3.3.1. Bending Length

Stiffness is the resistance of the material to external deformation and drape, which is also referred to as the bending rigidity of a material. The stiffness of the fabric was measured using the bending strip method, where a higher bending length refers to a higher stiffness. Bending length is the length a fabric can stretch to before bending under its own weight [56]. Thus, fabrics offering longer bending lengths are stiffer and have lower drape. Figure 6a shows that sample M is less flexible than the other nonwovens prepared due to its higher stiffness resulting from the entanglement of several layers. The higher stiffness of sample M is also related to its higher thickness. The prepared nonwovens showed higher flexibility and lower stiffness due to a relatively lower level of inter layer/inter fiber entanglements. The sample D showed approximately 24.36–58.62% higher stiffness as compared to the other samples developed due to the surface coating and modification via the formation of a nanofibrous network. The network of nanofibrous silicon between the cotton fibers enhanced the fabric’s stiffness [56,57].

#### 3.3.2. Tear Strength

The force needed to propagate a rip/tear in the nonwovens is called tear strength. Nonwovens are made of entangled fibers kept together by thermal, chemical, or mechanical bonding, which affects their tear strength. Figure 6b depicts the tear strength of the samples and shows that sample D exhibited maximum tear strength which is about 14.67% and 68–89.33% higher than the market sample M and the other nonwoven samples prepared, respectively. This is due to the surface modification using a silicon coating and nano fibers. The surface treatment strengthened the fabric and made it more difficult to be torn [58]. The other samples showed relatively lower tear strengths as the only resistance to tearing is offered by the inter-fiber friction. Sample M has multiple layers, so the relatively higher entanglement of fibers resulted in a comparatively higher tear strength than the other untreated nonwoven samples (A, B, and C).

### 3.4. Acoustic Characteristics

**Figure 7:**
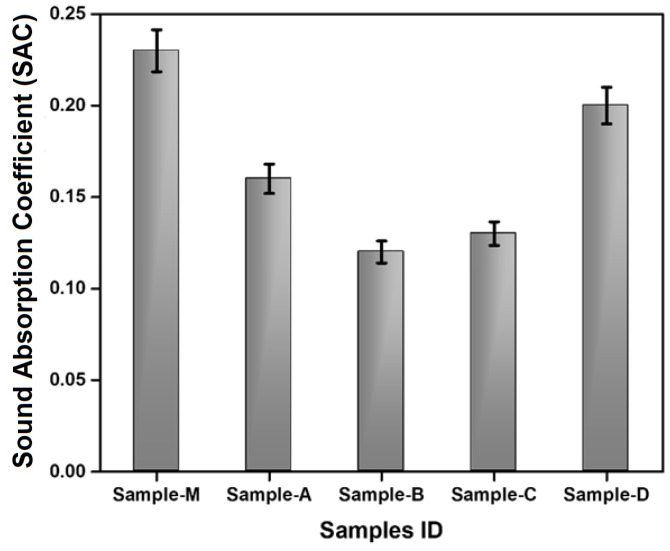
Acoustic characteristics of the nonwoven fabrics.

Sound-absorbing nonwoven materials are widely used for noise reduction. The sound absorption coefficient, which corresponds with frequency and direction, reflects the average absorption of normally incident sound waves. All the prepared nonwoven samples had the capability of sound absorption even with relatively lower thicknesses as shown in Figure 7. The values correspond to normal incidence absorption coefficients averaged over frequency.

The surface modification caused the pores to become blocked and the formation of silicon nanofibrous structures which increased air flow resistivity and thus the sound absorbed. The literature suggests that higher air flow resistivity is associated with higher sound absorption [58,59,60,61,62]. Low-frequency sound cannot penetrate easily through materials with lower porosity and air permeability. The main contributions to sound absorption at the mm pore scale are viscous friction and thermal exchanges at the pore walls. The narrower the pores compared to the viscous and thermal boundary layer thicknesses, the greater the energy loss from the penetrating sound waves. Similar findings were reported by other researchers [63,64,65,66,67]. The samples A, B, and C were porous and had relatively higher air permeability (lower resistivity) which caused lower sound absorption. Sample D has a 20–35% higher sound absorption compared to the other prepared nonwovens. This is attributed to the porous structure resulting from the possible formation of silicon nanofibers in between the cotton fibers in the sample. The extra absorption in these pores is related to molecular exchange and diffusion at pore walls. This might be a result of mass transfer and sorption processes happening in smaller pores [68,69].

The surface-modified sample D showed the most promising result that was comparable to the market sample M, which is already being used as a sound absorber.

### 3.5. Thermal Resistance

**Figure 8:**
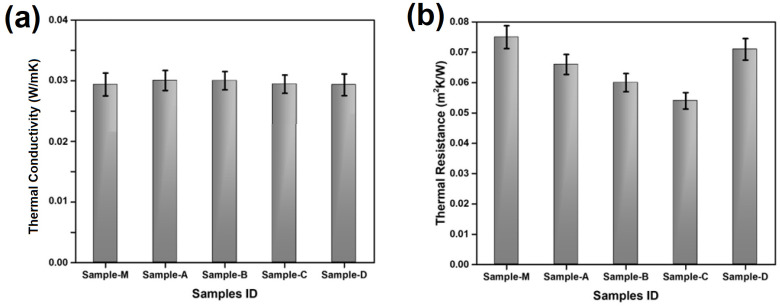
Thermal properties (**a**) Conductivity and (**b**) Resistance of the nonwoven samples.

The thermal conductivity was measured according to ISO 11092:2014 [40] using an Alambeta thermal insulation tester (Sensora, Liberec, Czech Republic). Results are shown in Figure 8a.

The thermal conductivity mainly depends on the type of fiber and the density of the material. Since they are composed of the same type of fibers, the conductivity was very similar. Sample D showed slightly lower thermal conductivity, which could be due to the silicon coating and nanofibers formed in the gaps between cotton fibers.

The thermal resistance of nonwoven materials refers to their capacity to hinder or impede the loss/transfer of heat through the material. Similar to acoustic behavior, thermal resistance increases as air permeability decreases (air flow resistivity increases). As shown in Figure 8b, sample M, which showed lower air permeability than other prepared nonwovens, also showed a lower thermal conductivity and hence a higher thermal resistance. The surface-modified sample D showed comparable results with the market sample M as its surface treatment made it thermally resistive by decreasing air permeability. The resistance is also enhanced by the formation of nano fibers between the cotton fibers. Sample D showed a 7–25% higher thermal resistance than the other nonwoven samples prepared. Since the micro air pockets formed in the treated and commercial samples are similar, the thermal performances of both samples were quite comparable. The thickness plays a vital role in achieving the desired thermal insulation [70,71,72].

### 3.6. Self-Cleaning, Non-Wetting, and Antibacterial Characteristics

**Figure 9:**
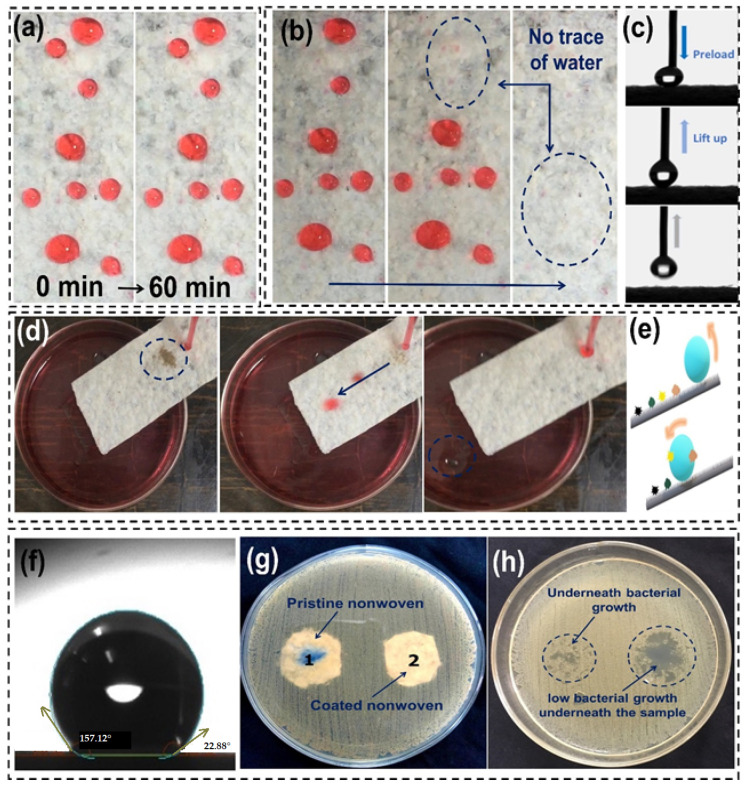
Functional characteristics of surface-modified nonwoven fabric. (**a**) Pronounced resistance to wetting (water colored chrome red). (**b**) Direct water stream interacting with the silane-treated nonwoven fabric. (**c**) Dynamic water repulsion displayed by the superhydrophobic surface. (**d**) Diagram showcasing the self-cleaning mechanism. (**e**) Water droplets glide over the fabric, effectively removing the scattered sand and mud mixture, a testament to the fabric’s hydrophobicity and surface texture. Notably, the droplet disintegrates after departing from the surface. (**f**) Water contact angle. (**g**,**h**) Antibacterial analysis of nonwoven fabric with and without silane treatment.

Surface hydrophobicity depends on chemical composition and geometric roughness. Figure 9 shows the performance of the self-cleaning, wetting, and antibacterial characteristic.

As shown in Figure 9a, the super hydrophobicity of surface-modified fabric sample D is significant. It resists the absorption of water and water flow and enables a self-cleaning effect. Various quantities of water droplets were applied on the superhydrophobic nonwoven fabric sample D, and they were not absorbed over a long period. Even after 1 h, droplets could be removed without trace, depicting the excellent water resistance. Figure 9b shows the phenomena with dotted circles where no wettability was observed after water droplets were removed. It shows non-wetting or the super hydrophobicity of the surface. The contact angle of the fabric surface was measured to be 157°, as shown in Figure 9f, whereas, after surface abrasion, it decreased to 151°, showing the high stability of the proposed superhydrophobic surface even after wear and abrasion. To assess the durability, a water jet impact was performed. High-speed water may damage hydrophobic coatings on surfaces.

Figure 9c shows the strong water-repellency of the fabric sample by rolling a droplet off without leaving residue. This is because superhydrophobic nonwoven fabric has poor interaction with/absorption of water droplets. Figure 9d shows a tilted fabric sample with a striking water stream. Even after 30 min of impact, the cloth resisted water without wear. This is an indication of a tough silicon treatment, responsible for its resilience. Superhydrophobic materials stay clean, decreasing the frequency of washing.

A mixture of sand and mud (as a solid contaminant) was dropped onto the fabric to test its physical self-cleaning effects against dust and dirt. As seen in Figure 9d, water droplets rolled off at a 6-degree angle, carrying the solid particles along during slide-off. As illustrated in Figure 9e, the nano structured surface roughness of the fabric enables it to be efficiently self-cleaning. The Cassie–Baxter condition of droplets on superhydrophobic surfaces explains their outstanding water repellent properties [73]. Finally, superhydrophobic coatings may be anti-icing. These surfaces are stable and water-repellent, so water droplets will retract or bounce off them, avoiding ice formation, and providing anti-icing capabilities.

The antibacterial properties or resistance to microbial growth of the nonwoven fabric extends their life and usefulness in several contexts. Figure 9g,h show the comparison of bacterial growth under the nonwoven samples with or without silicon treatment. It can be observed that the silicon-treated fabric significantly restricted the growth of bacteria underneath the sample, while pristine nonwoven was full of bacterial colonies and did not restrict their growth. This effect is due to the excellent water resistance/repellence of silicon nanofiber enabled superhydrophobic cotton nonwovens. Absence of water, which is essential component for microbial growth leads to antimicrobial (anti-fungal and anti-bacterial) performance.

## 4. Conclusions

Nonwoven fabrics, especially when subjected to surface treatments, display a range of enhanced properties, from increased strength to super hydrophobicity. These advancements render them appropriate for diverse applications and ensure their significance in contemporary textile innovations. In assessing the physical characteristics of nonwoven fabrics composed of 100% recycled cotton, with and without facile silicon treatment, various insights emerged during this research. The commercial sample M, obtained from the market, was thicker, multilayered, had relatively lower porosity, and large fiber entanglements. However, the silicon treated sample D had higher thickness and compactness due to the silicon nanofibers that sealed its pores. The silicon treatment induced the formation of porous nanofibrous layers binding the cotton fibers in the nonwoven material. Sample D had decreased macro porosity owing to its surface treatment, whereas sample M had reduced porosity due to its entangled fibrous layers. The untreated nonwoven samples showed higher porosity owing to loosely distributed/compacted fibers and were more permeable due to the lower level of entanglement among the fibers and layers. Solid entanglements made sample M the least flexible when testing material stiffness. The surface modification in silicon treated sample D made it 25–60% stiffer than the untreated nonwoven samples we prepared. The silicon treated sample showed a 70–90% higher tear strength compared to untreated fabrics. Surface treatment strengthened the nonwoven fabric, preventing ripping/tearing. Due to its multilayered structure, sample M outperformed untreated nonwoven samples in terms of tear strength. In spite of their relatively lower thicknesses, all the nonwoven samples developed from cotton waste (CW) fibers exhibited efficient sound absorption. The surface treatment in sample D improved sound absorption and was comparable to the commercial sample M. Fabrics with lower air permeability (higher air flow resistivity) show efficient sound and heat insulation. Sample D had comparable results to the market sample and showed 7–24% higher thermal resistance, and 20–35% higher sound absorption, respectively, as compared to the untreated nonwovens. The silicon treated fabric was superhydrophobic, water repellent, and efficiently self-cleaning. Finally, adding antibacterial characteristics can expand the life and utility of such nonwoven textiles.

Coating a natural hydrophilic material with a synthetic hydrophobic product certainly improved some properties, but some other pristine properties might be lost. Therefore, the properties related to its utility as a sound absorber and thermal insulator was studied. As was observed, the bending rigidity as well as tear strength were improved. The contact angle and antimicrobial properties were enhanced. Enhancing the micro-roughness of fabric via silicon nanofibers induced an efficient resistance to water absorption and led to the development of inherent self-cleaning characteristics. The antibacterial capabilities observed in the optimized sample were due to the superhydrophobic nature. The findings showed that surface treatment protected the nonwoven material against bacterial development, which is essential for sanitary materials. In conclusion, surface-modified nonwoven fabric has potential physical, acoustic, thermal, and antimicrobial qualities that make it useful for a broad range of applications including home furnishing and construction applications.

## Figures and Tables

**Table 1:** Average span length of fibers extracted from cotton waste (CW).

Span Length	Average Value ± Standard Deviation (mm)
50% span length	11.15 ± 0.74
2.5% span length	22.95 ± 2.24

**Table 2:** Properties of RTV silicon.

Properties	Average Value/Unit
Density	0.710–0.90 g/cc
Viscosity	50.0–88.0 cP
Shore Hardness	13.0–20.0
Tensile strength	0.310–0.860 MPa
Elongation at break	15.0–18.0%
Thermal Conductivity	0.0159–0.0200 W/m.K

**Table 3:** The nonwoven samples and their description.

Sample Code	Sample Description
Sample-M	Commercial market sample
Sample-A	Variation in thickness of nonwoven
Sample-B	
Sample-C	
Sample-D	Silicon nanofiber coated nonwoven sample

**Table 4:** Physical characteristics (average ± standard deviation) of the nonwoven fabrics.

Samples ID	Sample Details	GSM (g/m^2^)	Thickness (mm)	Porosity	Air Permeability (m^3^/s/m^2^)
Sample-M	Market Sample	70 ± 2	2.20 ± 0.04	0.54 ± 0.01	970 ± 10
Sample-A	Varied in thickness	131 ± 4	1.22 ± 0.01	0.69 ± 0.01	1349 ± 11
Sample-B		126 ± 4	1.14 ± 0.02	0.63 ± 0.01	1593 ± 14
Sample-C		95 ± 3	1.10 ± 0.02	0.60 ± 0.01	1290 ± 17
Sample-D	Surface modified Sample	142 ± 5	1.64 ± 0.04	0.45 ± 0.01	1090 ± 10

## Data Availability

Data are included in the article.
